# Complex interplay of evolutionary forces shaping population genomic structure of invasive Aedes albopictus in southern Europe

**DOI:** 10.1371/journal.pntd.0007554

**Published:** 2019-08-22

**Authors:** Verena Pichler, Panayiota Kotsakiozi, Beniamino Caputo, Paola Serini, Adalgisa Caccone, Alessandra della Torre

**Affiliations:** 1 Dipartimento di Sanità Pubblica e Malattie Infettive, Laboratorio affiliato Istituto Pasteur Italia—Fondazione Cenci Bolognetti, Università di Roma ‘Sapienza’, Roma, Italia; 2 Department of Ecology and Evolutionary Biology, Yale University, New Haven, Connecticut, United States of America; The Connecticut Agricultural Experiment Station, UNITED STATES

## Abstract

**Background:**

In the last four decades, the Asian tiger mosquito, *Aedes albopictus*, vector of several human arboviruses, has spread from its native range in South-East Asia to all over the world, largely through the transportation of its eggs via the international trade in used tires. Albania was the first country invaded in Europe in 1979, followed by Italy in 1990 and other Mediterranean countries after 2000.

**Methods/Principal findings:**

We here inferred the invasion history and migration patterns of *Ae*. *albopictus* in Italy (today the most heavily-infested country in Europe), Greece and Albania, by analyzing a panel of >100,000 single nucleotide polymorphisms (SNPs) obtained by sequencing of double-digest Restriction site-Associated DNA (ddRADseq). The obtained dataset was combined with samples previously analyzed from both the native and invasive range worldwide to interpret the results using a broader spatial and historical context. The emerging evolutionary scenario complements the results of other studies in showing that the extraordinary worldwide expansion of *Ae*. *albopictus* has occurred thanks to multiple independent invasions by large numbers of colonists from multiple geographic locations in both native and previously invaded areas, consistently with the role of used tires shipments to move large numbers of eggs worldwide. By analyzing mosquitoes from nine sites across ~1,000-km transect in Italy, we were able to detect a complex interplay of drift, isolation by distance mediated divergence, and gene flow in shaping the species very recent invasion and range expansion, suggesting overall high connectivity, likely due to passive transportation of adults via ground transportation, as well as specific adaptations to local conditions.

**Conclusions/Significance:**

Results contribute to characterize one of the most successful histories of animal invasion, and could be used as a baseline for future studies to track epidemiologically relevant characters (e.g. insecticide resistance).

## Author summary

Studying the genomes of invasive populations allows for the tracing of invasion processes. In this study, the authors have focused on the invasion of Albania, Italy and Greece by the Asian tiger mosquito, *Aedes albopictus*, one of the most successful invasive animal species worldwide, which in the last 40 years has invaded all continents resulting from the transportation of its eggs via human induced dispersion, especially the international used tire trades. Results showed that the invasion process was triggered by multiple independent (and most likely ongoing) importations of large number of eggs from different continents. Afterward, ground transportation of adult mosquitoes (e.g. by cars and trucks) and the capacity of the species to adapt to novel environmental conditions have allowed the colonization of whole countries, as clearly shown to have occurred in Italy. Overall, the results are consistent with a major role of human activities in the extraordinary expansion of the species, which today can be considered a permanent pest in all Mediterranean European countries, where it has already caused the infections of Europeans with exotic viruses (such as chikungunya) once confined to tropical regions and today representing a major concern also in temperate regions.

## Introduction

*Aedes (Stegomyia) albopictus* (Skuse, 1894)*—*commonly known as the Asian tiger mosquito or forest day mosquito—is one of the most successful invasive animal species worldwide [1]. In only a few decades this species has managed to spread from its native range in South-East-Asia all over the world, largely through the transportation of its relatively cold-hardy and long-lived eggs via the international trade in used tires [2,3].

The first evidence of this invasion dates to 1979, when *Ae*. *albopictus* was reported, for the first time outside its original range, in Albania [4]. Subsequently reports in Europe were made in 1990 and 1991, when it was found in north-west [5] and north-east Italy [6]. From there it spread rapidly, becoming established after only few years in most areas of the country <600m above sea level [7,8]. Today, Italy is the most heavily-infested country in Europe, with the highest densities in northern regions and in coastal areas of central regions [8]. Since 2000, it has spread to southern France, as well as to Greece, Spain, and the Balkan countries [9]. Since 2003 it has been repeatedly found in Switzerland (Ticino), probably as result of sporadic introductions from Italy [10], in the Netherlands at sites within importing bamboo companies [11,12], and in southern Germany along motorways, suggesting introduction by vehicles from southern Europe [13,14]. Similar sporadic positive collections have been also reported along motorways in Austria [15], Czech Republic [16], Slovakia [17], and the United Kingdom [18], although subsequent surveys were negative [19], suggesting that these introductions have not yet resulted in population establishments. However, this is not the case for other European locations where *Ae*. *albopictus* populations are established, including Slovenia (2007, [20]), Bulgaria (2011, [21]), Russia (2011, [22]), Turkey (2011, [23], and Romania (2012, [24]. Outside Europe *Ae*. *albopictus* was detected in the United States in Texas in 1985, where it was imported via a used tire shipment from Japan [25,26] and has since spread northward and eastward, facilitated by the movement of used tires along the interstate highways [26]. Now it is reported in at least 32 US states [27]. In Latin America it was first reported in Brazil in 1986 and later in Mexico in 1988 [28,29]. In Africa, it was first detected in 1990 in South Africa [30], but establishment was only reported in 2000 (Cameroon; [31]).

The worldwide invasion by *Ae*. *albopictus* was fostered by human activities via passive transportation of desiccated eggs by global and local used tire trade, and also the successive process of colonization was strongly influenced by humans. Similar to what had previously been observed for the closely related species *Aedes aegypti* [32–34], *Ae*. *albopictus* has possibly been able to progressively adapt to urban and sub-urban habitats [35], which offered abundant blood sources and anthropogenic larval breeding sites (such as rain catch basins and human-made containers e.g. tires, flower pots, cemetery urns etc.), substituting the natural larval habitats in its original range in south-east Asia (e.g. tree holes, bamboo stumps, and bromeliads). However, unlike *Ae*. *aegypti*, *Ae*. *albopictus* has been able to extend its range out of the tropics, mainly due to the development of diapausing eggs capable of overcoming cold periods and hatching only at the beginning of the following permissive season [36,37].

The widespread presence of an aggressive and day-time biting mosquito species such as *Ae*. *albopictus* in urban habitats in temperate countries not only represents a significant nuisance capable of changing human habits particularly in recreational areas [38,39], but also an increasing public health risk. The species is in fact a proven vector of dengue (DENV), chikungunya (CHKV) and zika virus (ZIKV) [40, 41] and is considered to be the major threat to public health among the invasive mosquito species in Europe [42]. Autochthonous cases of DENV and CHIKV vectored by *Ae*. *albopictus* have been recorded in the last 10 years in Europe, specifically in France [43–45] and in Croatia [46], and two autochthonous CHIKV outbreaks occurred in northeastern Italy in 2007 [47,48] and in central/southern Italy in 2017 [49, 50]. Modelling studies considering global warming projections and increasing urbanization suggest that *Ae*. *albopictus* will expand further towards northern Europe and Balkan countries, further extending the risk of tropical arbovirus transmission [8,51,52].

Genetic techniques can provide relevant information to infer routes and sources of invasive mosquito species, as well as the colonizing capacity, adaptability and behavior of invading lineages. However, *Ae*. *albopictus* human-mediated high dispersal ability makes it challenging to detect spatial structuring of genetic variation due to the high levels of gene flow [53]. The genetic studies carried out to date to reconstruct *Ae*. *albopictus* invasion dynamics and population history have revealed high levels of genetic connectivity among populations and the likely occurrence of repeated introductions, but were limited by the relatively low resolution of the genetic markers used (e.g. studies based on allozyme variations; [54–57]), the low levels of genetic variation among samples (e.g. studies based on mtDNA; [58–63]), and the low reproducibility and comparability among different datasets (studies based on microsatellites, [53, 59, 64–66]). Next-Generation Sequencing [NGS] approaches such as double-digest RADsequencing (ddRADseq, [67]) have been recently applied to overcome these limitations and reconstruct *Ae*. *albopictus* population history at finer geographic scales than formerly possible. Schmidt et al. [68] revealed previously undetectable evidence of isolation by distance and of human-mediated gene flow in an urban area in China, with distance from shipping terminals being the strongest predictor of genetic distance among mosquito urban populations. Sherpa et al. [69] detected a genetic footprint of isolation-by-distance among close (< 60 km) sites within Reunion island [Indian Ocean], as well as evidence for long-distance human-assisted transport to France. At a global scale, Kotsakiozi et al. [70] showed the existence of two major genetically differentiated clusters worldwide, each one including both native and invasive populations, confirming a scenario of multiple invasions found by previous studies and allowing for the inference of the geographic origin of several non-native populations.

We used the same ddRAD protocol as Kotsakiozi et al. [70] and combined data from that study with the ones collected in the present work to assess the distribution of *Ae*. *albopictus* genetic diversity along a ~1,000-km latitudinal gradient across Italy and in a few populations from neighboring European countries, which were invaded either earlier (Albania) or later (Greece) than Italy, and to infer their invasion history and migration patterns.

## Materials and methods

### Mosquito samples

*Aedes albopictus* samples analyzed in the present study originated from ovitrap collections carried out by local entomology teams from May to October 2016 in nine sites across Italy, one site in Albania and one in Greece (Table 1), At least five ovitraps were used in each site to avoid oversampling of siblings. Sampled eggs were sent to the Department of Public Health and Infectious Diseases (DPHID) at Sapienza University (Rome, Italy), where they were allowed to hatch. Larvae were reared to adults and emerged adults were preserved dry at -80°C for downstream analyses.

**Table 1:** Information on the 160 *Aedes albopictus* specimens included in the two datasets of the present study.

		country	sampling region	Range	N° of specimens	sampling year	code
*global dataset*	*2016-dataset*	Italy	Trentino—San Michele	invasive	7	2016	TN
			Veneto—Spinea	invasive	7	2016	VE
			Liguria—Imperia	invasive	7	2016	LG
			Emilia Romagna—Lido di Volano	invasive	7	2016	ER
			Marche—Ancona	invasive	7	2016	MA
			Lazio—Roma	invasive	7	2016	LZ
			Campania—Procida	invasive	7	2016	CA
			Puglia—Bari	invasive	7	2016	PG
			Sicilia—Messina	invasive	7	2016	SI
		Albania	Vlore	invasive	7	2016	AL
		Greece	Athens	invasive	7	2016	GR
	* *	Italy	Lazio—Roma	invasive	3	2005	ITA_2005
		Greece	NA	invasive	4	2013	GR_2013
		USA	Manassas, Virginia	invasive	4	2010	MAN
			Newark, New Jersey	invasive	4	2008	NEW
			Florida	invasive	6	2006	FLO
			Brownsville, Texas	invasive	4	2010	BRO
			Corpus Christi, Texas	invasive	4	2001	CORP
			Hawai	invasive	4	2006	HAW
		UK	Bermuda	invasive	4	2015	BER
		DRC	Kinshasa	invasive	4	2011	DRC
		Gabon	Franceville	invasive	4	2015	GAB
		Brazil	*Paqueta, Rio de Janeiro State	invasive	4	2015	PAQ
			Itacoatiara, Amazon State	invasive	4	2015	COAT
			Presidente Figueiredo, Amazon State	invasive	4	2015	PRES
			Salvador, Bahia State	invasive	4	2001	SALV
		Vietnam	Phu Hoa	native	4	2015	VTN
		Malaysia	Kuala Lampur	native	4	2006	KLP
		Singapore	Sentosa Island	native	4	2014	SIN
		Japan	Kagoshima	native	4	2008	KAG
			Tokyo	native	6	2008	TOK

Table includes information on the sampling region, site and year, the final number (N°) of individuals and the codes used as abbreviation for the sampling sites.

*specimens from Paqueta (Brazil) were not included in the study of Kotsakiozi et al. [70] but they were included in the present study to further clarify relationships between populations from Brazil and the native range.

Morphological species identification was performed according to Severini et al. [71]. For a subset of specimens (N = 16) morphological species identification was confirmed by DNA barcoding using a 710 bp fragment of the mitochondrial Cytochrome c oxidase subunit I (COI) gene and protocols described in Folmer et al. [72]. Sequencing of the PCR fragments was carried out at BMR s.r.l. (Padua, Italy). Comparison with available *Ae*. *albopictus* sequences for the same fragment (Barcode of Life Data System, [73]) confirmed the morphological species identification for the 16 specimens examined (sequence data are deposited to the GenBank database with accession numbers MK429679-MK429694).

To provide a broad spatial scale for some analyses we combined the newly collected data with those from Kotsakiozi et al. [70], and generated two data sets for global and within southern Europe analyses called hereafter *global-dataset* and *2016- dataset* respectively. The *global-dataset* included the *2016- dataset* of 11 populations from Italy, Albania, and Greece, along with populations from 5 native and 15 invasive collection sites in southern Europe, North America, South America, Africa, and the native Asian range of *Ae*. *albopictus* (Table 1). Sample size per locality (Table 1) varied from three (in the *global-dataset*) to seven (in the *2016-dataset*). Previous studies have shown that such a sample size is adequate for population genetic studies when analyzing large numbers of SNPs throughout the genome [74, 75]. Also, many population genetics studies based on thousands of SNPs have shown that such a sample size can be highly informative for inferring genetic diversity and differentiation in both vertebrates and invertebrates [76–78].

### DNA extraction and quantification

DNA extraction was performed on whole mosquito carcasses using the DNeasy Blood and Tissue kit (QIAGEN), according to the manufacturer’s instructions with two modifications: 1- the addition of 4ul of RNAse A/sample, and 2- DNA elution was carried out in double-distilled water (ddH2O). Quality of the extracted DNA was assessed by running 5ul on a 1.5% agarose gel stained with Midori Green Advance DNA Stain (Nippon Genetics Europe GmbH). DNA was quantified using a microplate reader and the Quant-iT PicoGreen kit (Thermo Fisher Scientific), according to manufacturer’s instructions. For the ddRAD library preparation only 88 specimens with high quality and molecular weight DNA were used.

### Double-digest Restriction site-Associated DNA [ddRAD] sequencing library preparation

The ddRAD libraries were prepared according to the Peterson et al. [67] protocol as modified by Gloria-Soria et al. [79]. Approximately 650 ng of DNA were doubled-digested using the NlaIII and MluCI (NEB) restriction enzymes in a total reaction of 50 ul, using an incubation at 37°C for 3 hours. Subsequently, individual barcoding was performed and Illumina indexes were added during a PCR amplification step (8 cycles). Sixteen barcoded and indexed specimens were multiplexed into each library. Size-selection was performed using the Blue Pippin electrophoresis platform (Sage Science), in order to select fragments of 215 bp of length as in [70], in order to be able to integrate our data with the previously published dataset. Libraries were sequenced (75bp paired-read sequencing) using the Illumina Hi-Seq 2000 platform at the Yale Center for Genome Analysis. To improve the sequencing quality, we increased the complexity of the sequencing lanes by spiking each library with another library constructed using different restriction enzymes.

### Sequence data processing

Sequence data (reads) were de-multiplexed using the *process_radtags* option of stacks [80] and mapped against the *Ae*. *albopictus* reference genome [81] using the paired option of Bowtie2 v.2.1.0 [82] and Samtools v. 1.3 [83]. Variant calling was performed using Samtools and filtering was carried out using the VCFtools v. 0.1.14.10 [84] obtaining a dataset, hereafter called *2016-dataset*, consisting of ~103K SNPs. The filtering details are provided in S1 Text.

Due to the relatively low quality of the assembled reference genome (consisting of 154,782 scaffolds with mean length of 201,000 bp, covering only ~2/3 of the estimated whole genome length; [81]), we also performed a “*de novo*” assembly for the raw reads (obtaining the *2016-denovo-dataset*), using *ipyrad* v.0.7.18 [85] to ensure that the quality of the reference genome did not affect our results. The filtering process as well as the analyses and the results of the *2016*-*denovo*-*dataset* are presented in the S1 Text.

To study the biogeographic relationships between the Italian, Greek, and Albanian samples and the worldwide ones, we concatenated the *2016-dataset* with the data from world-wide collections published by Kotsakiozi et al. [70] which has been produced following the exact same procedure described herein, obtaining a new dataset hereafter called *global-dataset*, consisting of ~38K SNPs from 31 sites. Details on the filtering process for the *global-dataset* are presented in the S1 Text.

Since some of the subsequent analyses (see Material and Methods section below) required the use of “unlinked” SNPs, we also created reduced SNP datasets by filtering each dataset for Linkage Disequilibrium (LD) estimates. For LD filtering we used the estimations and the same filtering process as described in Kotsakiozi et al. [70], using the *r* ^2^_max_/2 value as a threshold, where *r* ^2^_max_ is the maximum squared correlation coefficient value estimated by VCFtools. The LD filtered *2016-* and *global- datasets* consisted of ~55K and ~26K biallelic SNPs respectively.

The software PGDSpider v. 2.0.5.2 [86] was used to convert between file formats for downstream analyses, when necessary.

### Genetic diversity and differentiation and isolation by distance

Individual observed heterozygosity (H_obs) was estimated on the *2016-dataset* (~103K SNPs) using VCFtools [84]. Observed heterozygosity values were then grouped per population and a non-parametric Kruskal-Wallis (KW) test was used to compare the mean H_obs between populations. Expected heterozygosity (H_exp) per population was computed using the R-package adegenet 2.0.1 [87]. The same R-package was used to estimate significant differences of H_exp between populations by performing a permutation test with 500 simulations for each pairwise comparison. Inbreeding coefficients (F*IS*) per population averaged over all loci as well as the 95% confidence intervals (1,000 bootstraps) were estimated using the R-package hierfstat [88]. To compare the genetic diversity levels found at the fine spatial scale with those found at the global one, we also estimated the H_obs on the *global-dataset* (~38K SNPs), using the same software.

Genetic differentiation was estimated by computing F*ST* values between all pairs of populations of the *2016-dataset*, using Arlequin v.3.5 with 1,000 permutations and a significance level of 0.05. The partitioning of the genomic variation among and within populations was evaluated using a locus by locus hierarchical Analysis of MOlecular VAriance -AMOVA- [89], as implemented in Arlequin v.3.5.2.2, using 1,000 permutations.

Isolation by distance (IBD) was evaluated for the *2016-dataset* (~103K SNPs) through a Mantel test, using the R-package adegenet 2.0.1 [90], between a matrix of geographic distances and one of genetic distances between sampling sites (Nei’s genetic distance; [91]). Geographic distance was computed using the dist. function available in the R-package stats [92]. To evaluate the significance of the possible correlation between genetic and geographic distance, a randomization test with 1,000 random permutations of the two distance matrices was performed.

### Genetic structure

Maximum Likelihood (ML) and multivariate methods were used to assess the number of genetically distinct groups of populations present in the fine spatial scale, (i.e. the *2016-dataset*) and in the global scale dataset (*global-dataset*). The ML approach used was implemented in ADMIXTURE [93] on the LD filtered datasets (*2016-dataset*: 55,199 SNPs and *global-dataset*: 26,094 SNPs), as suggested by the authors. To avoid bias in cluster estimation due to the different sample size of the populations [94, 95] the *global-dataset* was pruned to retain approximately 4 specimens for each of the 31 populations used, totaling 123 samples. The best number of genetic clusters (K) was chosen based on the cross-validation procedure available in ADMIXTURE. Multivariate methods were also performed for the *2016-dataset* (~103K SNPs) and the *global-dataset* (~38K SNPs) and included a Discriminant Analysis of Principal Components (DAPC) and a Principal Components Analysis (PCA), implemented in R packages adegenet [96] and LEA [97], respectively. These multivariate approaches are valid alternatives to model based clustering approaches, because they do not require any assumption about underlying population genetic models [74, 96]. Here we used DAPC and PCA to complement our ADMIXTURE analysis on the genetic structure of our populations. To determine the number of significant components of PCA a Tracy-Widom test was performed following methods described by Patterson et al [74] and Frichot et al [97].

Because of the low genetic differentiation in the *2016-dataset* (see Results below), the number and composition of evolutionary clusters within this dataset was further explored using the program fineSTRUCTURE v. 2.0.7 [98] to investigate fine scale spatial patterns of genetic structuring across Italy, the main focus of the study. This method compares haplotype blocks across chromosomes and estimates shared co-ancestry among samples by dividing each haploid genome into stretches of DNA and identifying for each of these the most similar homologous stretch among the other specimens [98]. Thus, the method permits the identification of the other individual in the collection with the most similar ancestry for that part of the genome. This approach has been successfully used in several studies, including humans and Norway rats [76,99,100] to identify fine genetic structuring at small spatial scale [such as at the urban level; [100] and for populations showing very low genetic differentiation ([e.g. average F*ST* of 0.002; [99]). The fineSTRUCTURE algorithm has shown to outperform other clustering approaches (e.g. ADMIXTURE or STRUCTURE), especially when using high numbers of genetic markers [98]. Prior to the analysis, we pruned the LD filtered *2016-dataset* (55,199 SNPs) to retain only SNPs (4,094) from the longest scaffolds in which at least 25 SNPs per scaffold were present. This included scaffolds from 120,543–1,305,381bp length, representing ~4% of the *Ae*. *albopictus* genome. Data for each scaffold were phased using FASTPHASE v. 1.2 [101] and then converted to chromopainter, using a custom-made bash script. Initial analyses confirmed that our LD filtered dataset was effectively unlinked as indicated by the c-factor close to 0 (in our case of 0.026) computed by the software. This factor is related to the effective number of independent genetic elements present in the dataset and tends to 0 for completely unlinked data. The program fineSTRUCTURE was then run using the default parameters and the unlinked model.

### Migration analysis

To further investigate the dispersion events in the focal area of this study we analyzed gene flow patterns among *2016-dataset* samples after LD filtering (N SNPs = 55,199; N Inds = 77), by using the R-function divMigrate [102] implemented in the R-package diveRsity [103]. This approach computes migration rates between all sites and normalizes them afterwards to obtain relative migration rates which vary between zero and one. The software also searches for signs of significant asymmetry in gene flow direction between two sites by defining a hypothetical pool of migrants for each two sites combinations. This pool is then compared to the population pairs to calculate directional genetic differentiation and relative migration [102]. Computations were performed using the statistic introduced by Alcala et al. [104], which combines information from both genetic differentiation measures, Gst and D, to obtain estimates on the number of migrants per generation (Nm) and from that computes the relative migration rate. To evaluate the presence of significant asymmetric migration 1,000 bootstraps were performed.

## Results

### SNPs discovery

An average of 2,000,000 SNPs/specimen (SD = 316,533) was obtained after alignment of the *2016-dataset* (composed of 8 mosquitoes for each of the 11 European populations analyzed; Table 1) to the reference *Ae*. *albopictus* genome and filtering for a minimum mapping quality Q10. Out of the 88 specimens analyzed, 11 (one in each sample) were removed from downstream analyses because of poor sequencing quality (~10,000–20,000 reads/specimen compared to 3–10 million reads/specimens for the remaining ones). Thus, the final filtered (DP>7X, minimum coverage 70%, MAF 0.05) *2016-dataset* consisted of 103,289 SNPs and 77 individuals. The average number of SNPs per specimen was 86,000 (SD = 6,077) with an average depth of 25.5X (SD = 7.7). The amount of missing data was 16.4% (SD = 5.9) per specimen and 16.4% (SD = 8.2) per locus. After filtering this dataset for LD, a total number of 55,199 SNPs were retained. The same filtering strategy led to 38,550 SNPs for the *global-dataset* (composed of 160 mosquitoes from 31 populations; Table 1). The mean number of SNPs per specimen amounts to 34,215 (SD = 3,126) with an average depth of 23.1X (SD = 10.3) and an amount of missing data up to 11.2% (SD = 8.1) and 11.2% (SD = 5.5) per specimen and per locus, respectively. Filtering the *global-dataset* for LD, resulted in 26,094 SNPs. The results of the *de novo* assembly approach using ipyrad v.0.7.18 [85] on the *2016-dataset* are summarized in Table H in S1 Text. This analysis produced a final dataset of 20,805 SNPs.

### Genetic diversity and differentiation and isolation by distance

**Fig 1:**
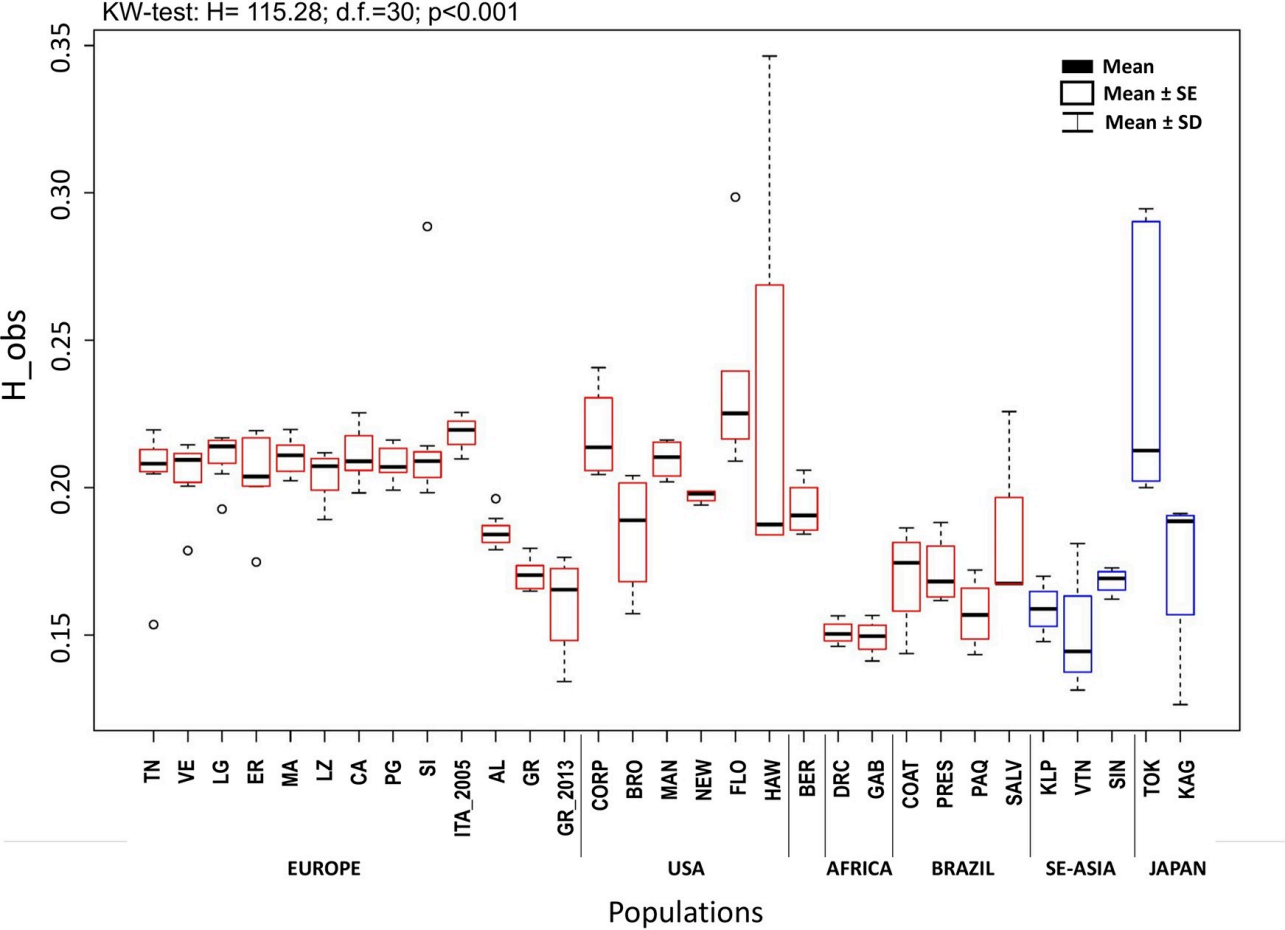
Individual observed heterozygosity averaged per population as estimated using vcftools for the *global dataset* of 38,550 SNPs. The mean, the standard deviation (SD), and the standard error (SE) are presented for each bar plot; dots represent outliers. The results for the non-parametric Kruskal-Wallis test, implemented to test for differences between the populations at a worldwide scale, are given above the graph. Color code: blue = native range; red = invasive range. Populations codes as in Table 1.

A comparison of the genetic diversity observed in Italy, Albania, and Greece (*2016-dataset*: 103,289 SNPs, Table 2) and worldwide (*global-dataset*: 38,550 SNPs, Table F in S1 Text) is shown in Fig 1. Although significant variation is observed among sites, no significant differences in heterozygosity (*p-*value = 0.464) are observed among the Italian sampling sites. These sites display significantly higher H_obs and H_exp levels than those observed in the geographically close Albanian (AL) and Greek (GR) sites (*p-*values <0.05; Table A in S1 Text) as well as lower frequencies of private alleles than the two non-Italian sites (range within Italy = 0–23; GR = 138; AL = 80; Table 2). Estimates of inbreeding are significantly higher for the Greek population (GR; F*IS* = 0.350) than for the Italian and Albanian ones. Among the Italian sites, significantly lower F*IS* values were found for Puglia (PG = 0.194) and Liguria (LG = 0.200) (Table 2) ones.

**Table 2:** Basic diversity statistics computed for the *2016-dataset* of 103,289 SNPs.

		private subst. sites	H_exp	H_obs	F*IS*	F*IS* CI	
**ITALY**	**TN**	7	0.218	0.176	0.293	0.286	0.296
	**VE**	1	0.216	0.182	0.263	0.259	0.267
	**LG**	23	0.209	0.19	0.200	0.194	0.203
	**ER**	6	0.217	0.183	0.259	0.254	0.263
	**MA**	0	0.228	0.19	0.267	0.263	0.270
	**LZ**	0	0.222	0.182	0.287	0.281	0.290
	**CA**	7	0.215	0.191	0.230	0.224	0.232
	**PG**	17	0.208	0.192	0.194	0.188	0.198
	**SI**	2	0.223	0.196	0.230	0.225	0.233
	**AL**	80	0.195	0.167	0.260	0.250	0.258
	**GR**	138	0.182	0.141	0.350	0.337	0.347

H_exp = expected heterozygosity, H_obs = observed heterozygosity, F*IS =* inbreeding coefficient with 95% confidence interval (F*IS* CI). A non-parametric Kruskal-Wallis test was implemented to test for differences between the populations at a European/Italian scale, with following results

KW- test statistics: within Europe: adjusted H = 33.48; d.f. = 10; *p*-value<0.001

within Italy: adjusted H = 7.70; d.f. = 8; *p-*value = 0.463

Estimates of genetic differentiation calculated on all the sampling sites in the *2016-dataset* (103,289 SNPs) are shown in Table B in S1 Text. F*ST* estimates range from 0.011 to 0.132 and are all significantly differentiated (*p*-values<0.05), with the exceptions of the two pairwise comparisons, including two sites from central Italy (MA and LZ: F*ST* = 0.011) and one of these sites (MA) from the Sicilian one (MA and SI, F*ST* = 0.024; *p-*values >0.05 after 1,000 permutations). The highest F*ST* values within Italy are observed between the Puglia samples and those from Veneto (F*ST* = 0.096) and Liguria (F*ST* = 0.094).

AMOVA results based on the *2016-dataset* (103,289 SNPs) are shown in Table C in S1 Text. The samples were divided into five groups according to geographic proximity: group1 = Albania (AL); group2 = Greece (GR); group3 = northern Italy (TN, VE, ER, and LG), group4 = central Italy (MA, LZ, and CA), group5 = southern Italy (PG and SI). Most of the genetic variance is explained at the individual level (68.1%), with only 3.55% and only 5.7% explained between groups and populations, respectively.

While we did not find indications for IBD when using the *global-dataset* (*p*-value = 0.753), the results on the *2016-dataset* (103,289 SNPs) suggest genetic differentiation with geographic distance (*p-*value = 0.001). This result is strengthened by focusing only on the 9 Italian sites (*p*-value = 0.012) and when excluding the Sicilian sample from this analysis (p-value = 0.001; Fig B in S1 Text), given its odd clustering with the northern Italian samples (see below).

The corresponding analyses for the *2016-denovo-dataset* produced data similar to those described above and are summarized in Table B, Table I and Fig H in S1 Text.

### Genetic structure and migration

**Fig 2:**
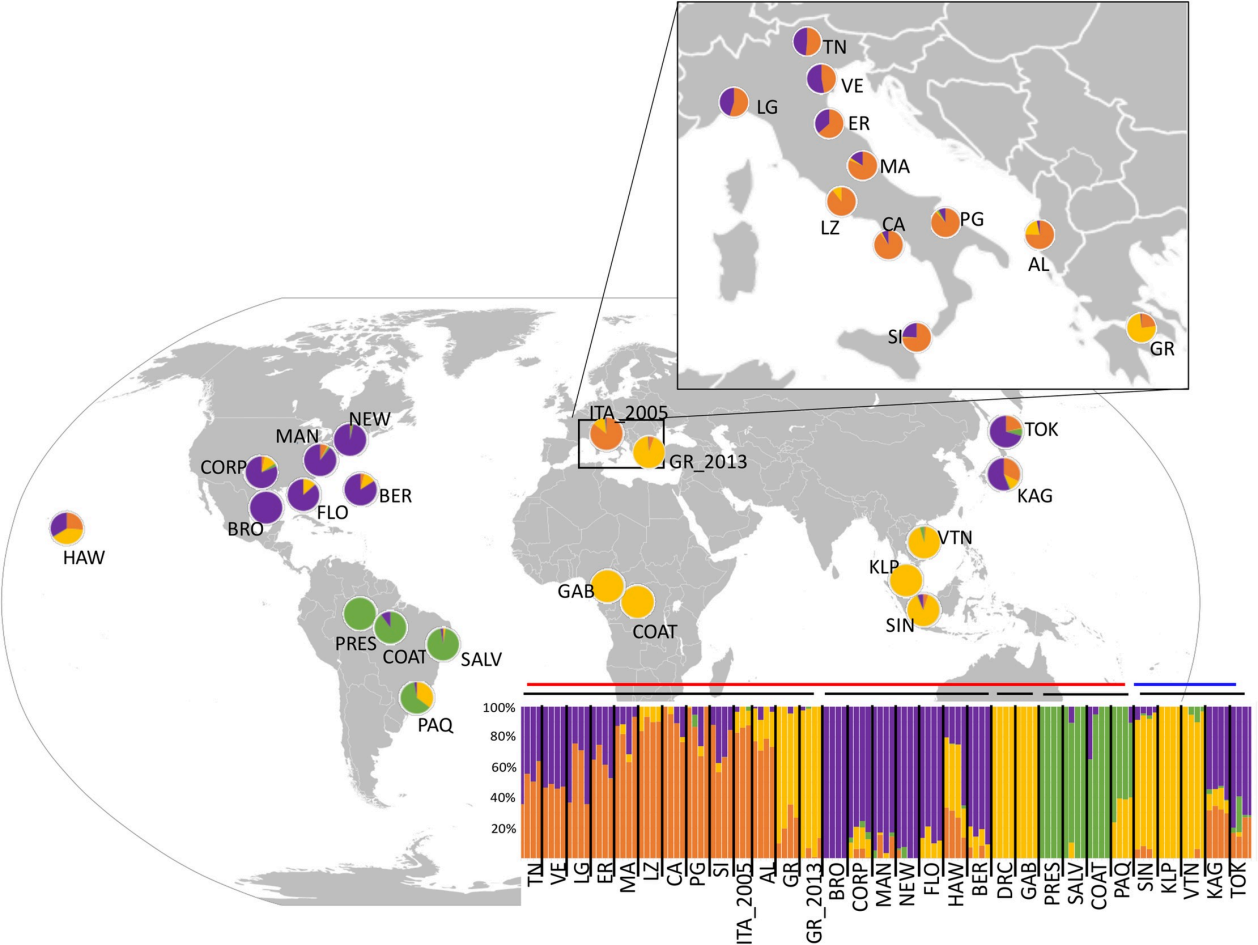
ADMIXTURE results obtained for the *global dataset*. Map (modified from https://commons.wikimedia.org/wiki/Atlas_of_the_world) shows mean ADMIXTURE Q-values, represented as pie charts for each sampling site, for best the clustering K = 4 for 123 specimens that were part of the LD filtered *global dataset* (N = 26,094 SNPs). Below the worldwide map the ADMIXTURE barplot obtained for the same set of specimens with individuals represented as vertical bars along the plot grouped by sampling sites (X axis) separated by vertical black bars. The Y axis represents the ancestry (Q value) with different colors representing the four clusters recovered by the analyses. Populations are encoded as in Table 1. Horizontal bars above the barplot indicate invasive (red bar) and native (blue bar) populations as well as to populations from the same continent (black bars). Populations codes as in Table 1.

The ADMIXTURE analysis for the LD filtered *global-dataset* (SNPs = 26,094) supports the existence of four genetically distinct clusters (K = 4; Fig 2), with the second-best clustering being three clusters (K = 3; Fig C in S1 Text). Mosquitoes from 15 out of the 31 sampling sites do not show evidence of genetic admixture (Q-values >0.80). However, clear signs of admixture (Q-values < 0.80 for all the specimens) are present in the Japanese (orange-purple), Albanian (orange-yellow), Hawaiian (orange-yellow-purple), one Brazilian (PAQ; green-yellow), and in several Italian sites, especially from the northern regions (i.e. TN, VE, LG, ER; orange-purple, Fig 2). The DAPC and PCA analyses for the *global-dataset* (SNPs = 38,550; Fig D, Fig E and Table G in S1 Text) are consistent with the spatial structuring identified by the ADMIXTURE analysis.

**Fig 3:**
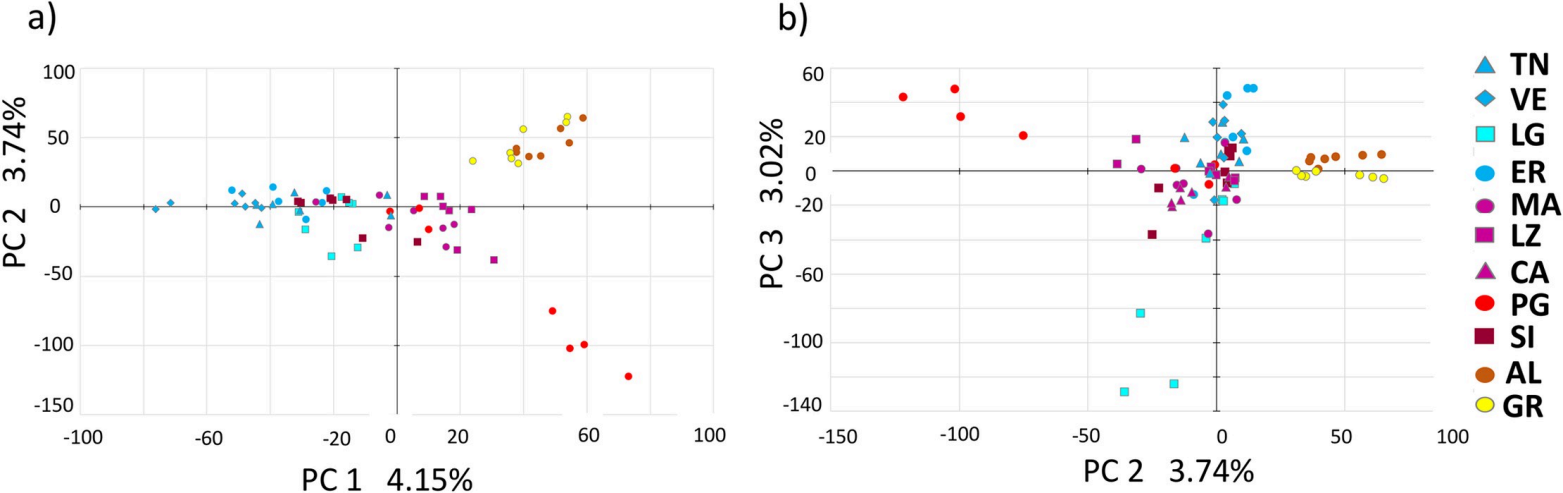
**Principal Components Analysis (PCA) presenting the projection of the *2016- dataset* (N = 77) on the first two PCs (a) and PCs 2 and 3 in (b).** Populations are encoded by different forms and colors according to a north-south gradient for Italy (north-Italy = light-blue, central-Italy = purple, South-Italy = red). Population codes as in Table 1.

While the ADMIXTURE analysis on the LD-filtered *2016-dataset* (55,199 SNPs) does not detect genetic structure (K = 1 supported as the best clustering), the DAPC analyses on the *2016-dataset* (103,289 SNPs), separates the samples in 8 groups (Fig A and Table E in S1 Text), confirming the differentiation among mosquitoes from Italy, Greece, and Albania as suggested by previous analyses. In addition, it also identifies 9 specimens from two Italian sites from southern (Puglia, PG: N = 4) and northern Italy (Liguria, LG: N = 5) as genetically distinct from all the other Italian specimens. The results of the PCA analysis (Fig 3) confirm the differentiation of the Italian specimens from the Albanian (AL) and Greek (GR) ones, and the genetic distinctiveness of at least 7 of the 9 individuals from LG (N = 3) and PG (N = 4) identified in DAPC analysis (Fig 3B). The PCA analysis also shows a latitudinal differentiation across Italy, not identified by the DAPC analysis, although 5 specimens from Sicily (SI) cluster with mosquitoes from northern, rather than southern, sites. Although the first few axes of the PCA analysis explain a small percentage of variance (11%), this is a common phenomenon when analyzing large sets of markers [76, 105]. Moreover, the Tracy-Widom -test confirms the significance of structure observed on the first 4 axes (p-value<0.0001; results including the fourth axis are not shown since they were similar to the ones using the first three axes). The clustering and multivariate analyses results for the *2016-denovo-dataset* are summarized in S1 Text and are largely consistent with the ones form the *2016-dataset* (Figs F and G, Table J in S1 Text).

**Fig 4:**
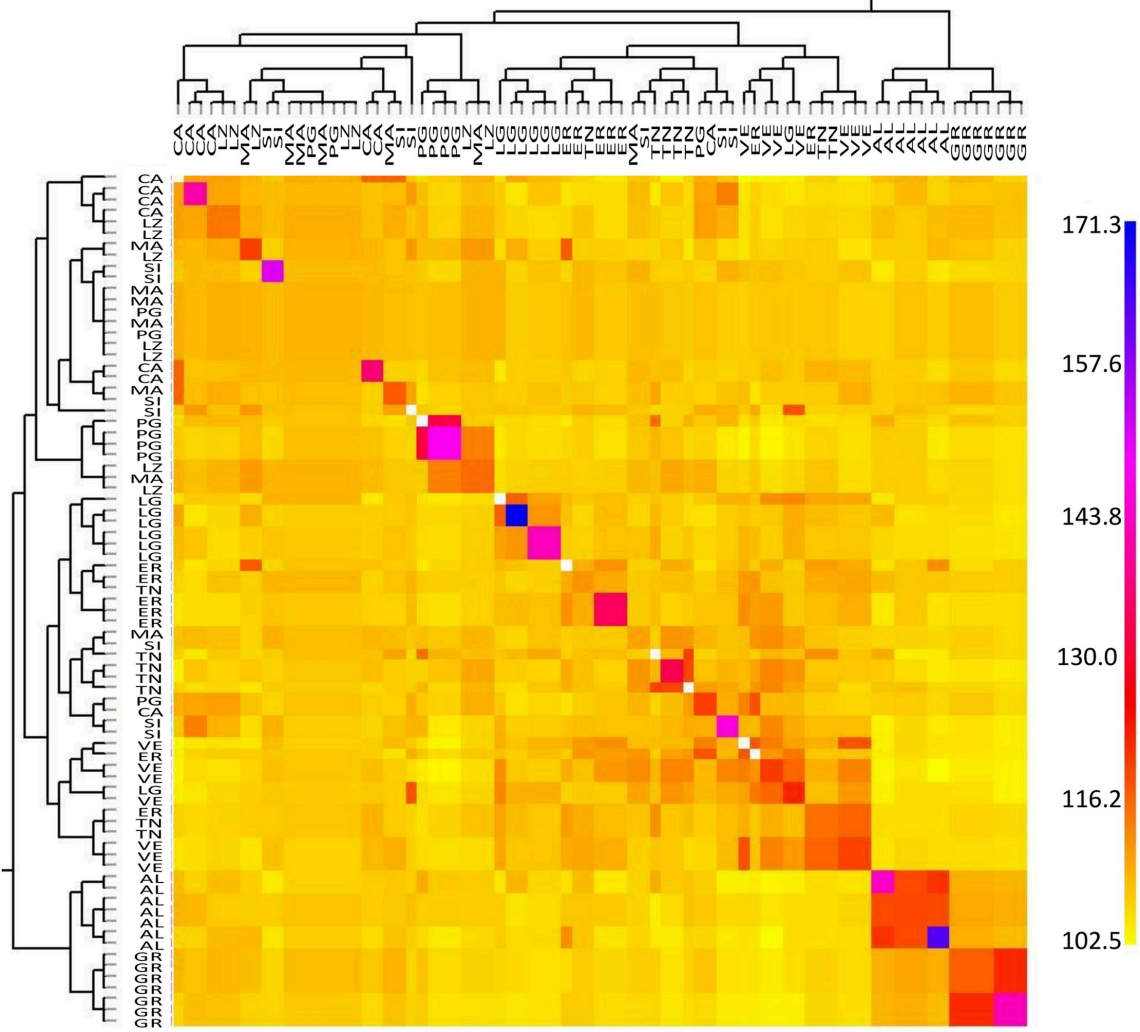
Co-ancestry heat map for the *2016-dataset* (N SNPs = 4,094). Heat map was obtained using the unlinked model in fineSTRUCTURE. The heat map shows the number of shared genetic chunks between the individuals. Co-ancestry scale is shown at the right with the lowest value in yellow and the highest in blue. The tree next to the heatmap shows the inferred relationships between the 77 specimens analyzed, with each tip corresponding to an individual. Populations are encoded as in Table 1.

To further investigate the possible presence of fine-scale genetic structure among the Italian populations, a fineSTRUCTURE analysis was implemented for the pruned *2016-dataset* (4,094 SNPs) only. Results (Fig 4), while confirming the differentiation between the samples from Albania, Greece and Italy, reveal structuring undetected by the previous analyses, as it separates the mosquitoes form the Italian sites into two groups. One group comprises samples from central and southern Italy sites (MA, LZ, CA, PG, and SI; N = 29). The other group includes mainly samples from northern Italy (TN, VE, LG, ER; N = 34), but also 6 specimens from central and southern Italy populations (1 from MA 1 from CA, 1 from PG, and 3 from SI). These specimens cluster also together with samples from northern Italy in the PCA analyses (Fig 3). However, they are not genetically distinct in the DAPC and ADMIXTURE analyses (Fig 2 and Fig A in S1 Text). Strongest co-ancestry within Italy is observed among the same specimens from PG (N = 4) and LG (N = 5) that form independent clusters in DAPC and, at least partially, in PCA-analyses.

**Fig 5:**
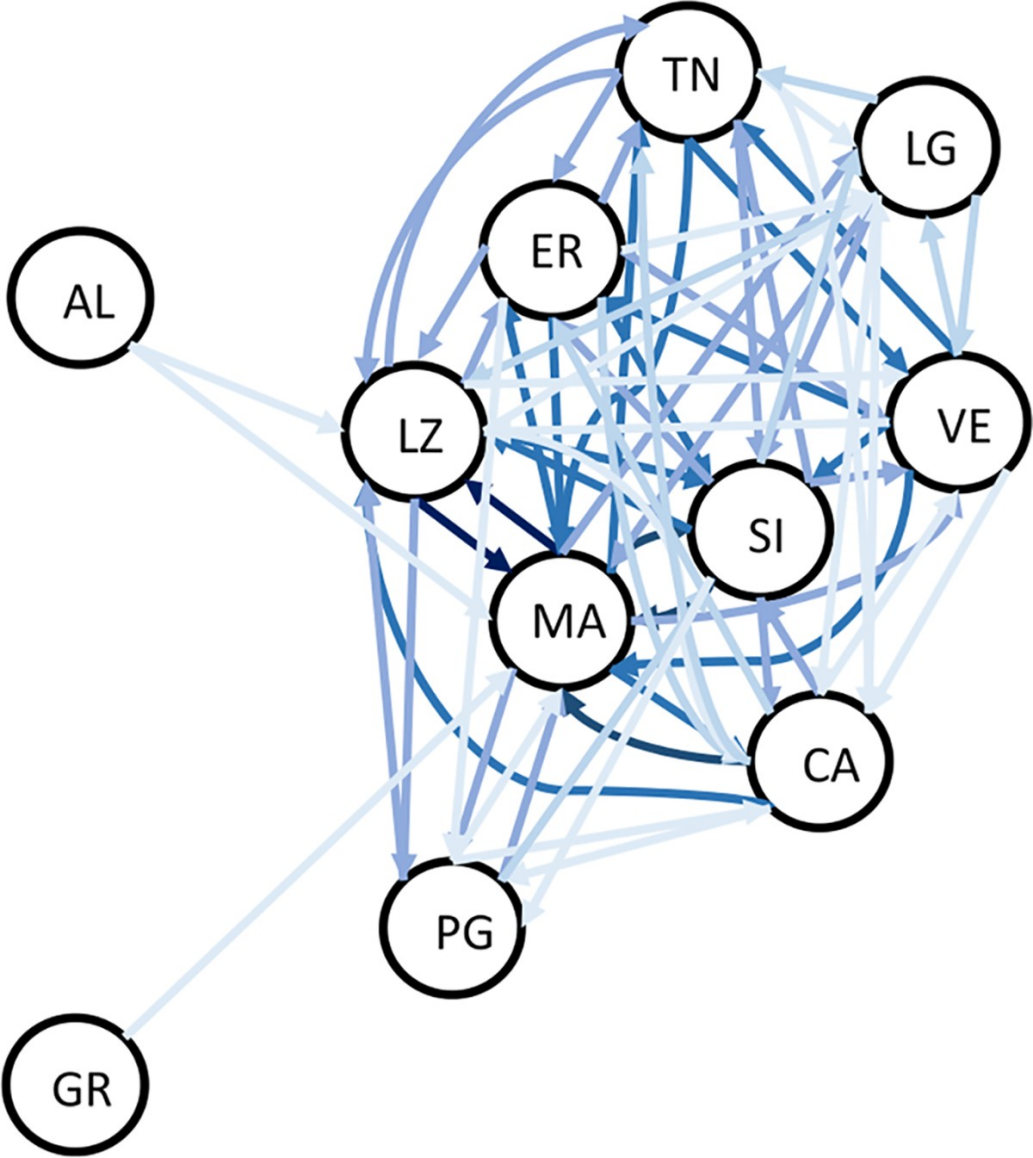
Relative migration network for the 2016-LD-filtered dataset (N SNPs = 55,199; N Inds = 77) obtained using the Nm [104] based estimate in the R-package divMigrate. Only migration rates>0.5 are represented, arrows indicate the direction of gene flow and arrow shading is relative to migration strength with darker shades representing higher values of relative migration. Populations are encoded as in Table 1.

Results of the divMigrate approach for the LD-filtered *2016-dataset* (SNPs = 55,199) reveal high levels of connectivity among the Italian sites (Fig 5, Table D in S1 Text) consistently with patterns identified by the estimates of genetic differentiation (F*ST*; Table B in S1 Text). Mosquitoes from most of the Italian sites results connected to each other in multiple ways, with those from Puglia (southern Italy) showing the lowest level of connection to the other Italian samples. As expected, the lowest levels of migration are observed for populations from Greece and Albania.

The migration analysis does not provide evidence of asymmetric migration. This is in contrast with what is expected if, after the initial invasion, range expansion occurred along a main latitudinal axis, as suggested by IBD analysis (Fig B in S1 Text). However, it is possible that the divMigrate analysis did not have the power to detect asymmetries because of the recent invasion and the low differentiation among populations, which makes it difficult to discriminate between old polymorphisms from the source population(s) and new ones.

## Discussion

The ddRAD approach applied allowed us for the identification of>100,000 SNPs with a very good coverage (>70%) and a limited percentage of missing data (16.4% ± 5.9 per specimen for the *2016-dataset*), which is a crucial feature for population genomic analyses, as several of the methods used to infer population genetic estimates are sensitive to missing data [106–108]. The high concordance between the results from the two *2016-datasets* (reference genome assembly and *de novo* assembly) allowed us to overcome the possible biases in SNP calling due to the low quality of the current *Ae*. *albopictus* assembly [81] strengthening the reliability of our inferences.

Slight discrepancies are present among results from different analytical method (ADMIXTURE, DAPC and PCA), likely due to technical limitations of the various methods in dealing with recently differentiated populations, which represents a challenge even when using thousands of SNPs, as in this study. It is also relevant to note that, despite the large number of genotype SNPs analyzed, the recent and fast global invasion makes the conclusive reconstruction of the history of the colonizing process by genetic data very hard. The following paragraphs comment the most interesting results obtained in view of current knowledge on *Ae*. *albopictus* bionomics.

### Levels and patterns of genetic diversity worldwide

Genetic diversity levels do not show systematic differences between native and invasive samples at a global scale, as already suggested by Kotsakiozi et al. [70], but differently from Sherpa et al. [69]. This possibly reflects the colonization of new areas by a large number of propagules retaining the levels of diversity of the source population[s], as already proposed to explain the lack of a genetic signature of “founder effects” for the US *Ae*. *albopictus* invasive populations [57].

Results corroborate the overall pattern of worldwide differentiation observed by Kotsakiozi et al. [70], but also identify an additional genetic cluster occurring in the native range and in Europe (Fig 2). Native populations are genetically distinct, with the two northernmost samples from temperate areas (Japan: TOK and KAG) genetically differentiated from the samples from tropical regions in Southeast Asia (KLP, VTN, and SIN), as indicated by both clustering and multivariate analyses (Figs C-E and Table G in S1 Text). The Southeast Asia samples are more genetically homogeneous than the Japanese ones. These show mixed ancestry (in agreement with [65]), with at least three distinct genetic clusters (yellow, orange, and purple, Fig 2), while the southeast Asian samples are mostly included in a single genetic cluster (yellow).

Despite the relatively uneven sampling density, the analyses of the samples from the invasive range suggest a complex pattern of repeated invasions from multiple geographic locations from the native range. The mosquitoes from US (MAN, NEW, FLO, BRO, CORP) share a similar pattern of genetic admixture with the Japanese ones (TOK, KAG), suggesting a northern Asia origin for the continental North American invasion. On the other hand, the Hawaiian sample (HAW) shows different relative frequencies of the three genetic clusters found in the mainland US samples. This could be due to multiple factors, including multiple colonization events or different colonization dynamics with genetic drift and reduced gene flow facilitating the establishment in this remote location of a genotypic fingerprint different than the one from the mainland populations. Mosquitoes from the two African sites (GAB, COAT) are genetically homogeneous, belonging to a single genetic cluster found mostly in South-East Asia. Mosquitoes from South American sites (PAQ, COAT, PRES, SALV) are genetically distinct from all others and mostly belong to a genetic cluster only found at low frequencies in one sample from the native range (Vietnam, VTN), underscoring a limitation of this study due to the limited sampling of the native range.

### Levels and patterns of genetic diversity in southern Europe

At the European scale, our results provide insights on the origin of the *Ae*. *albopictus* invasion in this continent and enable the identification of previously unidentified genetic structuring. Despite most genetic variation among the samples from Italy, Greece and Albania is found at an individual and intra-population level (>90%, Table C in S1 Text), most sites show significant genetic differentiation (Table B in S1 Text), with the Greek and Albanian ones being the most differentiated. Interestingly, the Greek samples have the highest level of private alleles (Table 2), notwithstanding the close proximity with Albania and the intense maritime traffic to/from south-central Italy. Both the Greek and the Albanian samples also display lower levels of genetic diversity and higher inbreeding coefficients than the Italian ones (Table 2), which could be a causal reflection of different colonization dynamics by *Ae*. *albopictus* in these countries. In Italy, after the first record in 1990, the species spread explosively over the whole country [9,109]. On the other hand, the range expansion in Albania and Greece has not been as rapid, possibly due to differences in the ability of the invading propagules to adapt to local environmental conditions and/or bottlenecks [110]. Similar low levels of genetic diversity and inbreeding in Albania were also suggested by Sherpa et al. [69].

Different clustering and multivariate analyses on both the *2016-* and *global-datasets* identified a genetic structuring consistent with that suggested by diversity statistics (Figs 2, 3, 4 and Table 2, Fig A in S1 Text). The Italian and Albanian samples show genetic admixture with three genetic clusters, one of which was not identified by Kotsakiozi et al. [70] (orange, Fig 2) and is also found in the native Japanese samples. The Greek samples assign to only two of these distinct clusters, with the prevailing one being the cluster mostly found in South Asian sampling sites (yellow). This suggests the occurrence of at least two independent invasions, one from a northern Asian lineage which colonized Albania and northern Italy (likely via passive dispersion from the USA, [55,111, 112] and another from a Southern Asia lineage, which colonized Greece and is present (in minor proportions) also in central Italy (i.e. LZ). While the Albania invasion likely came directly from a yet unidentified source in southern China thanks to the intense and close commercial relationship between these countries in the 70s [4,65,113], the similar genetic make-up of the populations from northern Italy and US is compatible with historical records showing that the invasion was initiated from a US propagule [112]. This scenario is consistent with previous data which suggested that the Italian invasion derived by multiple sources [55,65,114] and proposed similar geographic areas as the source of the European invasions [65,70].

### Levels and patterns of genetic diversity in Italy

The analysis of multiple Italian samples across a ~ 1,000 km longitudinal transect reveals a complex pattern of genetic structuring, likely derived from multiple ongoing processes. First, we find high levels of genetic diversity (Fig 1) which, being comparable with the ones from the native range, suggest that these populations originated from either large propagules and/or from multiple ones. The occurrence of multiple invasions is also suggested by the finding of some individuals collected in two port cities in Liguria and Puglia (Fig 3), which cluster apart in some multivariate analysis (Figs 3, 4 and Fig A in S1 Text). This suggests their likely origin from a different un-sampled geographic source and highlights the inherent limitation of this kind of studies, which even when carried out on samples from many populations, may suffer by not having the potential source populations included in the analyses. Since recent studies suggested that genetic variability in the native range is still widely underestimated [59,66], the availability of additional information on the genetic structure among populations in South-East Asia will likely help to disentangle *Ae*. *albopictus* migration history.

Second, although similarly diverse and mostly genetically distinct, the mosquitoes from the Italian sites also show evidence of isolation by distance (Fig B in S1 Text), latitudinal differentiation (Fig 3B) and geographically structuring into two population clusters from northern and southern regions (Fig 4). Superimposed on this spatial structuring we also found different levels of genetic admixture (Figs 2, 3 and 4) supporting both short and long-range dispersal, which is further confirmed by the migration analysis results (Fig 5 and Table D in S1 Text). The overall results suggest that, by using a large amount of genetic markers in combination with different types of population genomics methods, we were able to detect the complex interplay of drift, IBD mediated divergence, and gene flow in shaping the very recent invasion and range expansion of *Ae*. *albopictus* in Italy. Compared to previous work this study provides strong support to the hypothesis of Italy having been invaded several times by different source populations and by large propagules suggested by other studies based on allozymes, mitochondrial DNA or microsatellites [56,65,114]. However, contrary to previous work, this is the first one where clear signs of IBD are detected also in *Ae*. *albopictus*, and where the high genetic diversity, and thus adaptive potential, of Italian populations is highlighted. IBD has been commonly detected in a related species, *Ae*. *aegypti* [115–117]. Probably the older colonization history of this species, which dates back to several hundreds of years ago [118], compared to the very recent invasion history of *Ae*. *albopictus*, made the detection of subtle genetic differentiation patterns easier in *Ae*. *aegypti* than in *Ae*. *albopictus* when using only a handful of markers. The use of thousands of SNPs in *Ae*. *albopictus* as in the current study made the identification of the IBD pattern possible.

## Conclusions

The evolutionary scenario emerging from our analyses complement the results of other studies [55,57,65,70] in showing that the extraordinary global expansion of *Ae*. *albopictus* range has occurred thanks to multiple independent invasions by a large number of colonists from multiple geographic locations in both native and previously invaded areas. This is fully consistent with the known role of used tires shipment to move large numbers of eggs worldwide [9,112]. Results also confirm the contribution of extensive genetic intermixing between samples from different genetic backgrounds to the observed levels of structuring.

The genotyping of specimens from multiple sampling sites across a ~1,000-km transect in Italy allowed to highlight clear evidence of multiple invasions. Indeed, its geographic and geological conformation (with ~7,500 kms of coast and numerous commercial ports) makes Italy more susceptible to multiple invasions than other Mediterranean countries, which have been invaded more recently than Italy (e.g. Spain [119], France [120]). Direct evidence of adult *Ae*. *albopictus* dispersal by car in Spain [121], support the hypothesis that after invasion by maritime transportation and local colonization, passive transportation of mosquito adults by ground transportations has played a critical role in the fast long-range dispersal within Italy. This has allowed multidirectional migration, admixture between genetic background from different source populations, enhanced genetic diversity and, with it, the possibility for the species of establishing across a wide range of ecological conditions. In fact, *Ae*. *albopictus* is found all over Italy from coastal areas to areas up to 600 m above sea level, despite diverse climatic and environmental conditions [9]. Finally, evidence of population structuring due to drift but also to isolation by distance suggest that, despite this high connectivity, some specific adaptation to local conditions may be occurring (e.g. with reference to diapause photoperiod mechanisms). Overall, the explosive spread of *Ae*. *albopictus* in Italy could reflect the effect of the combination of different genetic sources in favoring the spread and adaptation of a species. The Italian situation should thus act as a warning bell for other European countries, where the tiger mosquito is at the moment present at low densities and/or in limited areas, highlighting the need of continuous and coordinated efforts in identifying entry points as soon as possible and in blocking or at least limiting the arrival and spread of new propagules, which could bring with them novel genetic variation that may be of epidemiological relevance.

The possibility to combine data from different studies by analyzing a common set of thousands of variable SNPs (as we did in this case merging our data with those by Kotsakiozi et al. [70] will facilitate future studies to compare the species population structuring in Italy and in other Mediterranean countries in order to better understand the different invasion and colonization dynamics across Europe and beyond. Identifying the main source populations implicated in recurrent invasions, as well as main routes and means of passive dispersal, is not only an interesting exercise to better characterize one of the most successful history of animal invasion, but may also contribute to understand (and possibly prevent) epidemiologically relevant process, such as spreading of insecticide resistance. For instance, the different invasion histories here shown between Italian and Greek samples are consistent with the presence of different alleles conferring resistance to pyrethroids in populations from the two countries [122]. It is also interesting to note that the Greek population showed reduced susceptibility to permethrin even in the absence of large scale usage, suggesting that it may be originated by an already resistant source population [123]. This points to the need of monitoring and possibly stopping new introductions not only in *albopictus*-free regions, but also in already highly infested ones. In fact, importation of insecticide resistant specimens may rapidly allow the species to colonize areas with ongoing mosquito control activities and change the effectiveness of control methods. Moreover, genetic mixtures may create new and more adapted genotypes, as in the case of two permethrin-resistant alleles (G1016 and C1534, already reported to be present in Italy and Greece, respectively; 122), whose association was shown to lead to enhanced resistance in *Ae*. *aegypti* [124,125]. Finally, high admixture and migration within Italy reinforce the possible contribution of passive transportation of adult females by cars or other means of ground transportation (in addition to movement of human cases) in spreading arboviruses from the core of an outbreak, as it may have been the case during the 2017 chikungunya outbreak which spread from a coastal village 70-km south of Rome, to Rome itself and other municipalities not only in Lazio region [126].

## Supporting information

S1 Text Supplementary material. (DOCX)

S1 Appendix SNP data for *2016-dataset*. (RAR)

S2 Appendix SNP data for *global-dataset*. (RAR)
