# Supplementary material for: Complex interplay of evolutionary forces shaping population genomic structure of invasive Aedes albopictus in southern Europe
Source: PLoS Negl Trop Dis. 2019 Aug 22;13(8):e0007554. doi: 10.1371/journal.pntd.0007554 (PMC6705758; doi:10.1371/journal.pntd.0007554)
Supplement: S1 Text — (DOCX) [file pntd.0007554.s001.docx]

**S1 Supplementary Material: Table of Contents**

1. Sequence data processing for the reference genome alignment
2. Supplementary results for the *2016-dataset*

Table A: Simulated p-value after 500 simulations for comparison of expected heterozygosity values

Table B: Population pairwise FST estimated for European populations.

Table C: Analysis of molecular variance (AMOVA) performed in Arlequin v.3.5.2.2 on the *2016- dataset* (103,289 SNPs).

Table D: Relative migration rate estimates for the *2016-LD-filtered dataset* (N SNPs= 55,199; N Inds= 77)

Table E: Composition of DAPC groups obtained for the *2016-dataset*

Fig A: Discriminant Analysis of Principal Components (DAPC) for the *2016-dataset*

Fig B: Isolation by distance represented as scatterplot

1. Supplementary results for the *global-dataset*

Table F: Individual observed heterozygosity averaged per population as estimated for the *global-dataset* of 38,550 SNPs using VCFtools

Fig C: ADMIXTURE barplot obtained for the *global-dataset* for second best K=3

Table G: Composition of DAPC groups obtained for the *global-dataset*

Fig D: Discriminant Analysis of Principal Components (DAPC) for the *global-dataset*

Fig E: Principal Components Analysis (PCA) for the *global-dataset*

1. *De novo* assembly of the *2016-dataset* using ipyrad

Sequence Data processing

Table H: Reads and clusters obtained from the de novo assembly of the *2016-dataset* as estimated by ipyrad

Genetic Differentiation

Table I: Analysis of molecular variance for the *2016-denovo-dataset*

Genetic Structure

Table J: Composition of DAPC groups obtained for the *2016-* *denovo-dataset*

Fig F: Discriminant analysis of principal components (DAPC) for the *2016-* *denovo-dataset*

Fig G: Principal Components Analysis (PCA) for the *2016-* *denovo-dataset*

Isolation by Distance

Fig H. Isolation by distance for the *2016-* *denovo-dataset* represented as scatterplot

1. *References*
2. *Sequence data processing for the reference genome alignment*

After alignment of the *2016-* and the *global- datasets* to the reference genome [1] as described in the main text, unmapped reads and reads of very low mapping quality (below Q10) were removed. Variant calling and filtering was performed using VCFtools v. 0.1.14.10 [2] and the following parameters: for the *2016-dataset* biallelic SNPs with minimum genotype depth (minDP) >7.0X, 70% coverage of the samples, minor allele frequency (MAF) of 0.05 while for the *global-dataset* minDP >5.0X and 80% coverage of the samples was used. Both datasets were filtered for Linkage Disequilibrium (LD) since the use of “unlinked” SNPs was a pre-requisite for some of the subsequent analyses. The LD filtering was performed using the –indep-pairwise option of PLINK 1.9 [3] with a window size of 100 variant counts, 50 as step and the r2max/2 value as a threshold, based on the preliminary results on the LD estimates of *Ae. albopictus* as described in Kotsakiozi et al [4].

1. *Supplementary results for the 2016-dataset*

| **Table A. Simulated p-values after 500 simulations for comparison of expected heterozygosity values** among the European populations. Values in bold identify significant comparisons. Population codes as in Table 1. |
| --- |
|  |

**Table B: Population pairwise FST estimated for European populations.**

Below the diagonal values obtained for the reference genome alignment for the *2016- dataset* (N=103,289 SNPs) and above diagonal F*ST* values obtained for the 2016-denovo dataset (N = 20,805 SNPs). Analyses were performed in Arlequin v.3.5.2.2. as described in Material and Methods section in the main text. Values in italic represent non-significant differentiation (*p*>0.05).

|  | **TN** | **VE** | **LG** | **ER** | **MA** | **LZ** | **CA** | **PG** | **SI** | **AL** | **GR** |
| --- | --- | --- | --- | --- | --- | --- | --- | --- | --- | --- | --- |
| **TN** | - | 0.025 | 0.059 | 0.041 | 0.029 | 0.037 | 0.053 | 0.074 | 0.038 | 0.086 | 0.096 |
| **VE** | 0.034 | - | 0.059 | 0.036 | 0.034 | 0.065 | 0.071 | 0.085 | 0.038 | 0.112 | 0.111 |
| **LG** | 0.067 | 0.069 | - | 0.074 | 0.049 | 0.064 | 0.066 | 0.086 | 0.054 | 0.106 | 0.111 |
| **ER** | 0.041 | 0.042 | 0.078 | - | 0.042 | 0.049 | 0.060 | 0.079 | 0.051 | 0.104 | 0.106 |
| **MA** | 0.031 | 0.046 | 0.051 | 0.044 | - | *0.012* | 0.027 | 0.040 | *0.023* | 0.074 | 0.081 |
| **LZ** | 0.041 | 0.078 | 0.072 | 0.051 | *0.011* | - | 0.030 | 0.038 | 0.037 | 0.073 | 0.083 |
| **CA** | 0.059 | 0.080 | 0.075 | 0.063 | 0.022 | 0.032 | - | 0.060 | 0.040 | 0.088 | 0.090 |
| **PG** | 0.078 | 0.096 | 0.094 | 0.079 | 0.040 | 0.041 | 0.061 | - | 0.065 | 0.099 | 0.114 |
| **SI** | 0.041 | 0.042 | 0.057 | 0.044 | *0.024* | 0.040 | 0.038 | 0.066 | - | 0.098 | 0.099 |
| **AL** | 0.097 | 0.120 | 0.121 | 0.105 | 0.070 | 0.074 | 0.090 | 0.110 | 0.094 | - | 0.110 |
| **GR** | 0.110 | 0.129 | 0.132 | 0.112 | 0.081 | 0.087 | 0.102 | 0.124 | 0.099 | 0.117 | - |

**Table C:** **Analysis of molecular variance (AMOVA) performed in Arlequin v.3.5.2.2 on the *2016- dataset* (103,289 SNPs).**

| **Source of variation** | **Percentage variation** | **Average F-statistics** |
| --- | --- | --- |
| among groups | 3.55 | F*CT* = 0.036** |
| among populations within groups | 5.70 | F*SC* = 0.059** |
| among individuals within populations | 22.68 | F*IT* = 0.319** |
| within individuals | 68.07 | F*IS* = 0.250** |

**=highly significant (*p*<0.001) after 1023 permutations

**Table D: Relative migration rate estimates for the *2016-LD-filtered dataset*** **(N SNPs= 55,199; N Inds= 77)**

Migration rates were obtained using the Nm based estimate in the R-package divMigrate, from (rows) and to (columns) European sampling regions.

|  |  | **TO** | | | | | | | | | | | |
| --- | --- | --- | --- | --- | --- | --- | --- | --- | --- | --- | --- | --- | --- |
|  |  | **TN** | **VE** | **LG** | **ER** | **MA** | **LZ** | **CA** | **PG** | **SI** | **AL** | **GR** |  |
| **FROM** | **TN** | - | 0.739 | 0.535 | 0.698 | 0.761 | 0.648 | 0.550 | 0.493 | 0.690 | 0.407 | 0.354 |  |
|  | **VE** | 0.762 | - | 0.558 | 0.755 | 0.742 | 0.523 | 0.514 | 0.478 | 0.736 | 0.361 | 0.321 |  |
|  | **LG** | 0.559 | 0.565 | - | 0.547 | 0.695 | 0.571 | 0.511 | 0.447 | 0.619 | 0.378 | 0.324 |  |
|  | **ER** | 0.661 | 0.697 | 0.508 | - | 0.745 | 0.663 | 0.563 | 0.517 | 0.703 | 0.404 | 0.358 |  |
|  | **MA** | 0.701 | 0.661 | 0.628 | 0.704 | - | 0.940 | 0.747 | 0.633 | 0.856 | 0.477 | 0.432 |  |
|  | **LZ** | 0.664 | 0.519 | 0.545 | 0.681 | 1.000 | - | 0.722 | 0.647 | 0.727 | 0.483 | 0.422 |  |
|  | **CA** | 0.559 | 0.511 | 0.513 | 0.584 | 0.821 | 0.785 | - | 0.539 | 0.687 | 0.425 | 0.369 |  |
|  | **PG** | 0.480 | 0.426 | 0.431 | 0.474 | 0.675 | 0.680 | 0.514 | - | 0.553 | 0.370 | 0.320 |  |
|  | **SI** | 0.648 | 0.666 | 0.593 | 0.682 | 0.855 | 0.728 | 0.673 | 0.552 | - | 0.431 | 0.375 |  |
|  | **AL** | 0.439 | 0.383 | 0.385 | 0.452 | 0.539 | 0.528 | 0.436 | 0.405 | 0.456 | - | 0.358 |  |
|  | **GR** | 0.407 | 0.373 | 0.363 | 0.426 | 0.515 | 0.490 | 0.425 | 0.374 | 0.441 | 0.378 | - |  |

| **Table E. Composition of DAPC groups obtained for the *2016-dataset*** (N inds= 77; N SNPs= 103,289). Population codes as in Table 1. |
| --- |
| **** |

| **Fig A.** **Discriminant Analysis of Principal Components (DAPC) for the *2016-dataset*** (N inds= 77; N SNPs= 103,289). The 8 groups identified by DAPC analysis (see Table S2) are shown in different colors. Population codes are as in Table 1. |
| --- |
|  |

| **Fig B.** **Isolation by distance represented as a scatterplot** of genetic vs. geographic distances for the *2016- dataset* for the Italian specimens after exclusion of the SI population. Right: histograms represent permuted values (i.e., under the absence of spatial structure) while the original value of the correlation between the distance matrices is represented by the dot. Significant spatial structure is suggested by the original value being situated out of the distribution of the permuted values. *p*-value=0.002 | |
| --- | --- |
|  | 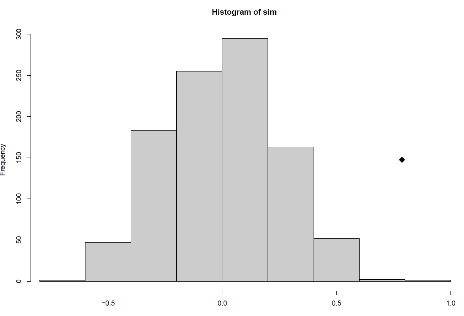 |

1. *Supplementary population structure results for the global-dataset*

| **Table F. Individual observed heterozygosity (H_obs) averaged per population for the *global-dataset*** (N inds= 160; N SNPs= 38,550). as estimated using VCFtools. Populations are encoded as in Table 1. |
| --- |
|  |

| **Fig C.** **ADMIXTURE barplot** **obtained for the 123 specimens part of the *global-dataset*** for the second best clustering suggesting K=3 (N =26,094 SNPs). Individuals are presented as vertical bars along the plot grouped by sampling sites (X axis) separated by vertical black bar. The Y axis represents the ancestry (Q value) with the different colors representing the three clusters recovered by the analyses. Populations are encoded as in Table 1. Horizontal bars above indicate invasive (red bar) and native (blu bar) populations as well as to populations from the same continent (black bars). |
| --- |
|  |

| **Table G. Composition of DAPC groups obtained for the *global-dataset*** (N inds= 160; N SNPs= 38,550). Population codes as in Table 1. |
| --- |
| **** |

| **Fig D.** **Discriminant Analysis of Principal Components (DAPC) for the *global-dataset*** (N inds= 160; N SNPs= 38,550). The graph represents the individuals as dots and the 15 groups identified by DAPC analysis as inertia ellipses. Different DAPC groups are shown in different colors and composition in population is roughly given in the color legend, while details are given in Table S3. A barplot of eigenvalues for the discriminant analysis (DA eigenvalues) is displayed in the inset. The number of bars represents the number of discriminant functions retained in the analysis and the eigenvalues correspond to the ratio of the variance between groups over the variance within groups for each discriminant function populations. |
| --- |

| **Fig E.** **Principal Components Analysis (PCA)** **presenting the projection of the *global-dataset*** on the first two PCs (N inds= 160; N SNPs= 38,550). Populations are shown in different forms and colors and encoded as in Table 1. |
| --- |
|  |

1. *De novo assembly of the 2016- dataset using ipyrad*

Sequence Data processing

To assure that the use of the reference genome [1] did not affect the quality of the alignment and as such our results, we also performed a *de novo* assembly approach using the *ipyrad* v.0.7.18 [5] on the newly produced *2016-dataset*.

For the *de novo* assembly (DN) pipeline we used ipyrad v.0.7.23 with the “pairddrad” option for the pair-end sequencing of the ddRAD data. Base calls with a phred quality score below 20 were converted to Ns (undetermined sites) and reads including more than four Ns were discarded. Based on the investigation for the *de novo* assembly parameters, as described in Kotsakiozi et al.[4], we set the clustering threshold to 0.90, the minimum depth (minDP) for statistical base call to seven, and the minimum coverage to 70% of the samples. All ipyrad parameters for which we do not mention any change here, were left as default, including the maximum number of SNPs per assembled locus set to 20 SNPs. The raw sequence reads were de-multiplexed and quality filtered using the same program. The software PGDSpider v. 2.0.5.2 [6] was used to convert between file formats for downstream analyses. To obtain the final *2016-denovo-dataset* used in the present analyses only loci situated on different clusters and thus considered “unlinked” were retained ending up with a final dataset of 20,805 SNPs. Details on the average number of reads, clusters, loci and depth per population obtained for the de novo assembly of the *2016-dataset* are presented on S5 Table.

Most of the analyses mentioned in the Materials and Methods section were performed also for the *2016-denovo-dataset* to assure that our results are stable and not affected by the quality of the reference genome used for the assembly. All results appear widely consistent with results obtained for the *2016-dataset* and are shown in Tables 3 (F*ST*-values in the main text), S6 (AMOVA), and S7 (DAPC clusters) and in Figs S6 to S8. ADMIXTURE analysis for the 2016-*denovo-dataset* produced the same result as for the *2016-dataset*, with K=1 as the best clustering and therefore no figure is presented

| **Table H.** **Reads and clusters obtained from the *2016-denovo-dataset* as estimated by ipyrad**. All values have been averaged per population and populations are encoded as in Table 1.  Abbreviations: reads_raw: number of reads obtained after demultiplexing the samples allowing no mismatch between the barcodes of the two reads (R1 and R2) per sample, reads_passed: number of reads that passed quality filtering, clusters: the number of clusters identified using the clustering threshold, clusters_high: the number of clusters that passed the mindepth thresholds requirement, loci: number of loci left in the final dataset after applying all the filtering parameters, depth: depth as averaged over all loci left in the final dataset. |
| --- |
| \| **Population** \| **reads_raw** \| **reads_passed** \| **clusters** \| **clusters_high** \| **loci** \| **depth** \| \| --- \| --- \| --- \| --- \| --- \| --- \| --- \| \| TN \| 18949962 \| 18801806 \| 2521740 \| 464833 \| 19936 \| 32.3 \| \| VE \| 15679586 \| 15557046 \| 2281335 \| 419286 \| 19490 \| 28.4 \| \| LG \| 13185383 \| 13083190 \| 2034783 \| 378589 \| 19285 \| 25.9 \| \| ER \| 14163186 \| 14045683 \| 2039176 \| 405593 \| 19547 \| 26.8 \| \| MA \| 12027388 \| 11937269 \| 1927637 \| 359821 \| 18560 \| 24.1 \| \| LZ \| 14998445 \| 14884421 \| 2171127 \| 402923 \| 19131 \| 27.8 \| \| CA \| 11410641 \| 11324441 \| 1884841 \| 321835 \| 17990 \| 25.2 \| \| PG \| 13601832 \| 13503452 \| 1953216 \| 377125 \| 18873 \| 26.8 \| \| SI \| 13267150 \| 13177910 \| 1937616 \| 382151 \| 19261 \| 26.3 \| \| AL \| 14285466 \| 14171567 \| 1861385 \| 405323 \| 19220 \| 27.3 \| \| GR \| 13778828 \| 13680060 \| 1921561 \| 422706 \| 17780 \| 24.9 \| |

Genetic Differentiation:

| **Table I. Analysis of molecular variance** (AMOVA) performed in Arlequin v.3.5.2.2 on the *2016-denovo-dataset* (20,805SNPs). Groups were defined as described in the Material and Methods section in the main text. |
| --- |
|  |

Genetic structure

| **Table J. Composition of the DAPC-groups obtained for the *2016-denovo-dataset*** (N inds= 77; N SNPs= 20,805). Populations codes as in Table 1. |
| --- |
|  |

| **Fig F.** **Discriminant Analysis of Principal Components (DAPC) of the *2016-denovo-dataset*.** The analysis performed on the 77 samples of the *2016-denovo-dataset* (N SNPs = 20,805) identified 8 DAPC-groups shown by different colors. The origin of the individuals in of the eight DAPC-groups is shown in the color code and details can be found in Table S6. The graph represents the individuals as dots and the DAPC-groups as inertia ellipses. A barplot of eigenvalues for the discriminant analysis (DA eigenvalues) is displayed in the inset. The number of bars represents the number of discriminant functions retained in the analysis and the eigenvalues correspond to the ratio of the variance between groups over the variance within groups for each discriminant function. Populations codes as in Table 1. |
| --- |
|  |

| **Fig G.** **Principal Components Analysis (PCA)** **presenting the projection of the *2016-denovo-dataset*** (N inds= 77; N SNPs= 20,805) on the first two PCs (A) and PCs 2 and 3 in (B). Populations are encoded by different shapes and colors according to a north-south gradient for Italy (north-Italy: blue; central-Italy: purple; South-Italy: red). Populations codes as in Table 1. |
| --- |
| A   |
| B   |

Isolation by Distance

| **Fig H.** **Isolation By Distance (IBD) represented as scatterplot** of Nei’s genetic distance vs. geographic distance for the *2016-denovo-dataset*  for the Italian specimens after exclusion of the SI population (N inds= 56; N SNPs = 20,805). Right: histograms represent permuted values (i.e., under the absence of spatial structure) while the original value of the correlation between the two distance matrices is represented by the dot. Significant spatial structure is suggested by the original value being situated out of the distribution of the permuted values. p-value after 1,000 simulations = 0.002 | |
| --- | --- |
|  |  |

1. *References*

1. Chen X-G, Jiang X, Gu J, Xu M, Wu Y, Deng Y, et al. Genome sequence of the Asian Tiger mosquito, Aedes albopictus, reveals insights into its biology, genetics, and evolution. Proc Natl Acad Sci U S A. 2015;112(44):E5907-15.

2. Danecek P, Auton A, Abecasis G, Albers C a., Banks E, DePristo M a., et al. The variant call format and VCFtools. Bioinformatics. 2011;27(15):2156–8.

3. Purcell S, Neale B, Todd-Brown K, Thomas L, Ferreira MAR, Bender D, et al. PLINK: a tool set for whole-genome association and population-based linkage analyses. Am J Hum Genet. 2007 Sep;81(3):559–75.

4. Kotsakiozi P, Richardson J, Pichler V, Favia G, Martins A, Urbanelli S, et al. Genomic insights into the recent worldwide invasion of the Asian tiger mosquito, *Aedes albopictus.* Ecol Evol. 2017.

5. Eaton DAR. PyRAD: Assembly of de novo RADseq loci for phylogenetic analyses. Bioinformatics. 2014;30(13):1844–9.

6. Lischer HEL, Excoffier L. PGDSpider: an automated data conversion tool for connecting population genetics and genomics programs. Bioinformatics [Internet]. 2012 Jan 15;28(2):298–9. Available from: http://dx.doi.org/10.1093/bioinformatics/btr642
